# Protein expression, characterization and activity comparisons of wild type and mutant DUSP5 proteins

**DOI:** 10.1186/s12858-014-0027-0

**Published:** 2014-12-18

**Authors:** Jaladhi Nayak, Adam J Gastonguay, Marat R Talipov, Padmanabhan Vakeel, Elise A Span, Kelsey S Kalous, Raman G Kutty, Davin R Jensen, Phani Raj Pokkuluri, Daniel S Sem, Rajendra Rathore, Ramani Ramchandran

**Affiliations:** Department of Pediatrics, Medical College of Wisconsin, CRI Developmental Vascular Biology Program, P.O. Box 26509, C3420, 8701 Watertown Plank Road, Milwaukee, WI 53226 USA; Department of Chemistry, Marquette University, Wehr Chemistry Building, P.O. Box 1881, 535 N. 14th Street, Milwaukee, WI 53201 USA; Department of Biochemistry, Medical College of Wisconsin, Children’s Research Institute (CRI) Developmental Vascular Biology Program, Translational and Biomedical Research Center, 8701 Watertown Plank Road, P.O. Box 26509, Milwaukee, WI 53226 USA; Center for Structure-based Drug Design and Development, Department of Pharmaceutical Sciences, Concordia University of Wisconsin, Mequon, WI 53097 USA; Biosciences Division, Argonne National Laboratory, 9700 S. Cass Avenue Lemont, Argonne, IL 60439 USA; Department of Obstetrics and Gynecology, CRI Developmental Vascular Biology Program, P.O. Box 26509, C3420, 8701 Watertown Plank Road, Milwaukee, WI 53226 USA

**Keywords:** DUSP5, Mutation, Vascular anomalies, Protein purification, Molecular modeling

## Abstract

**Background:**

The mitogen-activated protein kinases (MAPKs) pathway is critical for cellular signaling, and proteins such as phosphatases that regulate this pathway are important for normal tissue development. Based on our previous work on dual specificity phosphatase-5 (DUSP5), and its role in embryonic vascular development and disease, we hypothesized that mutations in DUSP5 will affect its function.

**Results:**

In this study, we tested this hypothesis by generating full-length glutathione-S-transferase-tagged DUSP5 and serine 147 proline mutant (S147P) proteins from bacteria. Light scattering analysis, circular dichroism, enzymatic assays and molecular modeling approaches have been performed to extensively characterize the protein form and function. We demonstrate that both proteins are active and, interestingly, the S147P protein is hypoactive as compared to the DUSP5 WT protein in two distinct biochemical substrate assays. Furthermore, due to the novel positioning of the S147P mutation, we utilize computational modeling to reconstruct full-length DUSP5 and S147P to predict a possible mechanism for the reduced activity of S147P.

**Conclusion:**

Taken together, this is the first evidence of the generation and characterization of an active, full-length, mutant DUSP5 protein which will facilitate future structure-function and drug development-based studies.

**Electronic supplementary material:**

The online version of this article (doi:10.1186/s12858-014-0027-0) contains supplementary material, which is available to authorized users.

## Background

The mitogen-activated protein kinases (MAPKs) pathways are activated by dual phosphorylation of tyrosine and threonine residues in their activation loops [1]. To regulate the MAPKs, proteins that dephosphorylate at serine/threonine and tyrosine residues have evolved. One class of phosphatases that dephosphorylate serine/threonine and tyrosine residues is dual specificity phosphatases (DUSPs) [2]. Our laboratory has previously identified one of the members of the DUSP family, DUSP5, as critical for vascular development and disease [3]. DUSP5 is well characterized to specifically interact and dephosphorylate phosphorylated extracellular regulated kinase (pERK), and does not act on other MAP Kinases, such as p38 and JNK [2,4,5]. Also unique to DUSP5 is that it localizes solely to the nucleus, and its expression is induced by active pERK signaling [2]. Therefore, DUSP5 acts as a negative feedback loop on active pERK signaling. It was also shown that DUSP5 is phosphorylated by pERK and can retain inactive ERK in the nucleus, thus providing another layer of tight regulation [5].

Structurally, DUSP5 is predicted to have two globular domains; an N-terminal ERK binding domain (EBD) and a C-terminal phosphatase domain (PD) [2,6]. A crystal structure of the DUSP5 PD [6] reveals a potential binding site for the phosphate of pERK, which was occupied by anionic sulfate groups. The PD domain is in part responsible for dephosphorylating pERK to ERK. Previously, we identified a mutation in DUSP5 that changed the serine to proline residue at the 147th position (S147P) in patients afflicted with vascular anomalies [3]. Vascular anomalies are inborn errors of developmental pathways, and are classified broadly into two groups, namely vascular tumors and vascular malformations. Vascular tumors such as hemangiomas appear after birth, rapidly grow within the first 6 months, and suddenly involute, resulting in scarring and other complications [7,8]. Vascular malformations, on the other hand, are present at birth and are sustained throughout the life of the individual, while affecting arteries, veins and lymphatics. The identified S147P mutation, which is uniquely located in the linker region adjoining the EBD, could potentially interfere with the dephosphorylating activity of the DUSP5 protein on its substrate pERK, and thus contribute to the inception and progression of vascular anomalies.

Originally thought to be purely a structural motif, we propose that the DUSP5 linker region plays an important role in DUSP5 binding with pERK. To assess whether S147P mutation has a consequence on DUSP5 activity, we generated a glutathione-S-transferase (GST)-tagged version of both wild type DUSP5 and mutant S147P proteins. Here, using biochemical assays and computational modeling, we have demonstrated that the S147P mutation results in reduced DUSP5 activity and describe a potential novel role for the linker region in pERK binding.

## Methods

### Cloning of GST-DUSP5 and GST-S147P constructs

The full-length wild type human DUSP5 cDNA was cloned in a heterologous expression system as GST-DUSP5 fusion protein using the PGEX-6P1 GST vector (GE Healthcare). The cDNA was PCR amplified using Phusion High fidelity DNA polymerase kit (F-530 L: Thermo Scientific). The PCR was performed with Phusion GC buffer (F-519) containing 3% DMSO on a purchased cDNA template (Cat. #: EX-Z2247-M49) from GeneCopoeia, Inc. USA. The primer sequences are: (hDUSP5-f: TTT*GAATTC*GCCACCATGAAGGTCACGTCGCTC hDUSP5-r: TTT*CTCGAG*GCAGGATGTGGCCGTTGCCAC). The gel purified PCR product was cloned in frame with GST in the pGEX-6P-1 vector at the *EcoRI* and *XhoI* restriction sites. A single clone was selected after sequencing for protein expression. Further, S147P mutation was introduced in the wild type GST-DUSP5 plasmid by following the kit protocol as described in the QuickChange Lightning Site-Directed Mutagenesis Kit (Agilent Technologies, USA). The primer sequences are: hDUSP5S147PF: GTGGATGTAAAACCCATTCCCCAAGAGAAGATTGAGAGT, and hDUSP5S147P: ACTCTCAATCTTCTCTTGGGGAATGGGTTTTACATCCAC.

### GST-DUSP5 and GST-S147P protein expression

GST-DUSP5 and S147P constructs were transformed into Rosetta2 cells (Novagen, 71402–3). Pre-culture was started with picking a single colony from an overnight grown plate. Ten mL of overnight culture was used to inoculate 1 L of terrific broth containing 10 mL of 40% glucose, 100 μg/ml carbenicillin and 34 μg/ml chloramphenicol. The culture was grown at 37°C with shaking at 250 RPM. When OD 600 reached 0.6, the culture was induced with 0.05 mM IPTG at 16°C for 20 h. The cells were then harvested by centrifugation with 5000 RPM for 15 min (Sorvall Super T21) at 4°C. The cell pellets were re-suspended in a lysis buffer containing 50 mM Tris HCl pH 8.0, 1% Triton X-100, 0.5 M NaCl, 10 mM EDTA, 10 mM EGTA, 10% glycerol, 2 mM PMSF, 5 mM DTT, 0.4 μg/ml Antipain and 0.2 μg/ml Leupeptin. The re-suspended cells were sonicated (Misonix, Sonicator 3000), and centrifuged at 10,000 RPM for 45 min (Sorvall) at 4°C.

### GST pull down assay

GST-DUSP5 was absorbed to 10 μl glutathione beads with shaking at 4°C for 2 h. After extensive washing, 20 μl of SDS loading buffer was added into the resulting beads. The mixture was heated for 3 min, loaded on 4-20% SDS-PAGE gels, and stained with Coomassie blue.

### GST-DUSP5 and GST-S147P protein purification

GST-DUSP5 and GST-S147P proteins were purified using affinity chromatography. The cell extract of both recombinant proteins were allowed to pass through pre-equilibrated glutathione sepharose 4B beads twice. The column was then washed thoroughly with lysis buffer and wash buffer A50 (containing 50 mM Tris–HCl pH 7.5, 50 mM NaCl, 5% glycerol and 1 mM DTT). The recombinant proteins were then eluted using elution buffer (20 mM glutathione + wash buffer, pH 7.5). The eluted protein was concentrated using centrifugal filters (Amicon Ultra# UFC905008, Eppendorf 5810R centrifuge).

### PreScission protease cleavage of GST-DUSP5 and GST-S147P

GST was cleaved off the proteins using PreScission protease enzyme (GE Healthcare, 27-0843-01). For every 100 μg of protein, 1 U of PreScission protease enzyme was used in 1x protease cleavage buffer containing 50 mM Tris HCl pH 7.5, 150 mM NaCl, 1 mM DTT and 5% gycerol. The mixture was incubated for 4 h at 4°C. The mixture was then allowed to incubate with GST beads for 30 min, and passed through the column. The resultant flow through contains cleaved protein.

### Dynamic light scattering

Dynamic Light Scattering (DLS) measurements at room temperature were performed on DynaPro instrument (Protein Solutions) with default parameters using a 12-μL quartz cuvette. Data were acquired and processed with the Dynamics (v5) software. Proteins (DUSP-5 at 1.5 mg/mL and S147P at 1.8 mg/mL) in buffer (25 mM Tris.Hcl, pH 7.5, 50 mM NaCl, 1 mM DTT, 20% glycerol) were centrifuged at 13,000 rpm for 5 minutes prior to measurement.

### CD spectroscopic verification of folded DUSP5

CD spectroscopy experiments were performed using a Jasco J-710 spectropolarimeter. Protein solutions were dialyzed into CD buffer containing 25 mM Tris–HCl pH 7.5, 50 mM NaF, 1 mM TCEP, 5% glycerol, and 0.01% IGEPAL. Protein concentrations used for CD were adjusted to 0.2 mg/ml and then placed in a round cuvette with a 1 mm path length. Data were collected over a wavelength range of 190–260 nm. At least five scans were acquired and the averages were used for final analysis. CD spectra were deconvoluted and secondary structure prediction obtained using DICHROWEB [9] and the K2d algorithm [10].

### Para-nitrophenol phosphate (pNPP) activity assay

Recombinant DUSP5 phosphatase activity was measured using *para*-nitrophenol phosphate (pNPP) as a synthetic substrate, and was performed as previously described [11] with modifications. Assays were performed in 96-well plates at a total volume of 200 μl. In brief, 1 μM of recombinant protein was added to assay buffer containing 100 mM Tris–HCl (pH 7.5), 100 mM NaCl, 5 mM MgCl_2_, 1 mM DTT, 0.5% Triton-X 100. The substrate, pNPP was added last to the wells at the indicated concentrations using a multichannel pipet. Absorbance was measured every 30 seconds on a micro-plate reader (Molecular Devices, SpectraMax, 340PC384) at 405 nm for 5 min, and the ΔAbs/Δt were plotted on Michaelis-Menten graphs with nonlinear regression best-fit curves using Prism 6.0 software (GraphPad Inc., San Diego, CA).

### ERK dephosphorylation assay

To conduct this assay, 10 ng of GST-tagged recombinant phosphorylated human ERK2 (R&D Systems, 1230-KS) was incubated with and without the indicated DUSP5 proteins (0.5 nM final concentration) for 5–15 min, as indicated. The reactions were halted with 2x Laemmli sample buffer and subjected to SDS-PAGE. The proteins were transferred to polyvinylidene difluoride (PVDF) and immunoblotted using antibodies to pERK (Cell Signaling Tech., #9106) and total ERK (Cell Signaling Tech., #9102). Bound antibodies were visualized using horseradish peroxidase-linked anti-mouse IgG (Cell Signaling Tech, #7076S) and anti-rabbit IgG (Cell Signaling Tech, #7074S), respectively, and ECL reagents (Pierce, 34708) according to the manufacturer’s protocol.

### Homology modeling of EBD

The ERK-binding domain of human DUSP5 (i.e. residues 1–140) was constructed with the YASARA Structure 13.9.8 software [12,13] using the ‘hm_build’ macro (see http://yasara.org/homologymodeling.htm for the detailed description of the homology modeling procedure). Interestingly, the top ten structures produced by YASARA were based on two templates, originating from the two crystal structures (PDB codes: 2A2K [14] and 1YMD [15]) of human Cdc25B phosphatase catalytic domain (17% identity). Besides creating the template-based models, YASARA also generated a hybrid model, in which it combines the best features of the template-based models and thereby increases the accuracy of resulting model beyond each of the contributors. We refined the hybrid model as well as the best template-based structures of EBD by performing 500-ps molecular dynamic simulations (see the details of MD below) and found that the hybrid model has the lowest energy. The credibility of this hybrid model, which was used in subsequent modeling studies, was further solidified by the fact that the most conserved residues were localized on one face of EBD (Additional file 1: Figure S4), as it is commonly found in various DUSPs [16,17]. It is noted that the above fully automated homology modeling procedure did not make use of the known structure of EBD of DUSP6 from NMR studies (23% identity, PDB code: 1HZM) [16]. To ensure the validity of our model, we deliberately performed homology modeling using the structure of EBD of DUSP6 and found that the resulting structure had a low Z-Score value (−4.0; Z-Score < −2 can be interpreted as poor, and < −4 as bad), when compared to the Z-Score of the hybrid model (−0.1). The confidence in our hybrid model was further ascertained by the Structure Analysis and Verification Server v4 (SAVES, see http://services.mbi.ucla.edu) and PROtein Structure Evaluation Suite and Server (PROSESS, see http://www.prosess.ca/) [18], and details of the analysis are compiled in Additional file 2: Figure S5.

### Molecular dynamic simulations

The molecular dynamics simulations were performed using Amber 03 force field [19] with a 7.86 Å force cutoff and the Particle Mesh Ewald algorithm [20] to treat long-range electrostatic interactions. A simulation cell was defined to be cubic with periodic boundary conditions in all directions, with a distance of at least 10 Å from protein along each axis. The simulation box was filled with TIP3P water [21] at density of 0.997 g/ml and Na/Cl ions, which were placed at the lowest/highest electrostatic potential locations to neutralize the cell and approximate the physiological saline solution (0.9% NaCl). The protonation states of acidic/basic amino acids were set according to *p*H = 7.4. To remove bumps and correct the covalent geometry, the structure was energy-minimized by a short steepest descent minimization, followed by simulated annealing (time step 2 fs, atom velocities scaled down by 0.9 every 10th step) until convergence was reached, i.e. the energy improved by less than 0.05 kJ/mol per atom during 200 steps. The obtained structure was used as a starting point for molecular dynamic simulations, which were performed at *T* = 298 K with time step of 1.25 fs for intramolecular interactions and 2.5 fs for intermolecular interactions. Atomic velocities were rescaled using the modified Broensted thermostat, based on time-averaged temperatures, which does not depend on the strongly fluctuating instantaneous microscopic temperatures [22].

### Ethical considerations

This research does not involve human subjects, human materials, or vertebrate animals. All experiments were conducted under the Medical College of Wisconsin institutional safety approval.

## Results

### Optimization of conditions for DUSP5 protein expression

**Figure 1:**
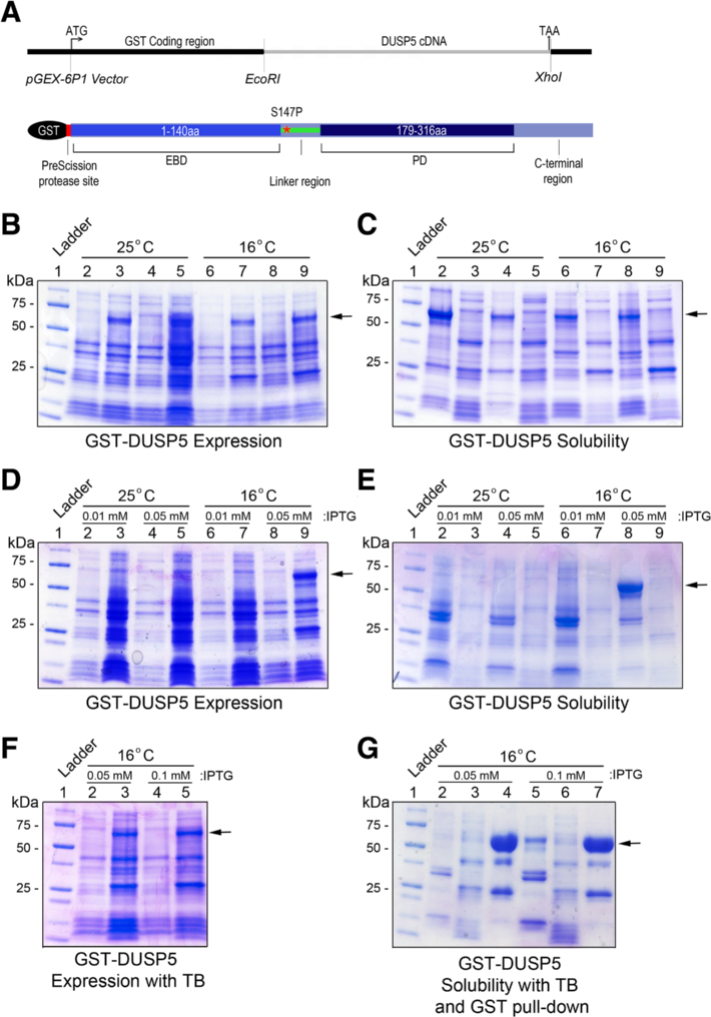
**Construction of plasmid and Optimization of expression condition. (A)** pGEX-6P-1 vector map. **(B-C)** Effect of temperature on GST-DUSP5 protein expression. **(B)** Lane 1: MW marker, lanes 2, 4, 6 and 8: cell pellet before induction, lanes 3, 5, 7 and 9: cell pellet after induction. Lanes 2–3, 4–5 are induced at 25°C for 20 h, lanes 6–7, 8–9 are induced at 16°C for 20 h. Lanes 4–5 and 8–9 are duplicate samples for lanes 2–3 and 4–5 respectively. **(C)** Effect of temperature on GST-DUSP5 solubility, Lane 1: MW marker, lanes 2 & 4 and 3 & 5 are cell pellet, and cell lysate post sonication, and samples induced at 25°C overnight for 20 h, lanes 6 & 8 and 7 & 9 are cell pellet and cell lysate post sonication, and samples induced at 16°C overnight for 20 h. **(D-E)** Effect of IPTG concentration on GST-DUSP5 protein expression. **(D)** Lane 1: MW marker, lanes 2, 4, 6 & 8: cell pellet before induction, and lanes 3, 5, 7 & 9: cell pellet after induction either with 0.01 mM or 0.05 mM IPTG at 25°C or 16°C for 20 h. **(E)** Effect of IPTG concentration on GST-DUSP5 solubility. Lane 1: MW marker, lanes 2, 4, 6 & 8 cell pellet post sonication, and lanes 3, 5, 7 & 9 are cell lysate post sonication for expression induced with either 0.01 mM or 0.05 mM IPTG, at 25°C or 16°C. **(F)** GST-DUSP5 protein expression using terrific broth and 0.05 mM and 0.1 mM IPTG and 16°C overnight induction. Lane 1: MW marker, lanes 2 & 4 and 3 & 5 are cell pellet before and after induction. Lanes 2–3: 0.05 mM IPTG, lanes 4–5: 0.1 mM IPTG. **(G)** GST-DUSP5 protein solubility and GST pull down. Lane 1: MW marker, lanes 2 & 5 are cell pellet post sonication, lanes 3 & 6 are cell lysate post sonication, lanes 4 & 7 are protein adsorbed on GST beads.

To generate full-length DUSP5 protein, our laboratory used an *E.coli* expression strategy to express the full-length human protein tagged with Glutathione-S-Transferase (GST), which enhances solubility of the protein. Previous attempt at generating soluble protein with histidine tag was not successful (data not shown). Generation of full length active and mutant DUSP5 proteins will greatly assist structural studies and the ability to discern the mechanism of action of these proteins in the progression of vascular anomalies. The full-length human DUSP5 was cloned into a pGEX-6P1 vector (Figure 1A) and DNA sequencing was performed to confirm the DUSP5 sequence. We transformed pGEX-6P1-DUSP5 into Rosetta 2 cells. Initially, temperature and IPTG concentrations were empirically optimized. Bacteria growth was tested in lysogeny broth (LB) under two different temperature conditions of 16°C and 25°C (Figure 1B). Under each condition we performed + and – IPTG (0.2 mM) for comparison purposes and lysed cells by sonication to separate the soluble and insoluble components as described in the materials and methods section. DUSP5 protein was observed by Coomassie staining but, unfortunately, the DUSP5 protein band was again observed in the insoluble cell pellet (Figure 1C). We next varied the IPTG concentrations using 0.01 mM and 0.05 mM, while maintaining the 16°C and 25°C temperature conditions. We detected the DUSP5 protein at 16°C, 0.05 mM IPTG, and overnight induction condition (Figure 1D). However, the protein was still observed in the insoluble cell pellet (Figure 1E). We next switched the media from LB to terrific broth (TB), and the expression was carried out at 16°C using an overnight induction with 0.05 mM and 0.1 mM IPTG concentration (Figure 1F). We observed a GST-DUSP5 protein band at the correct size in the soluble fraction when induced with at 16°C with 0.05 mM and 0.1 mM IPTG (Figure 1G, lanes 3 and 6). However, the protein was less soluble in the 0.1 mM IPTG condition as demonstrated by the presences of a strong protein band in pellet fraction (Figure 1G, lane 5). Based on the testing of various parameters, we concluded that TB media with 0.05 mM IPTG at 16°C was the best condition for generating soluble GST-DUSP5 protein.

### GST-DUSP5 protein purification

**Figure 2:**
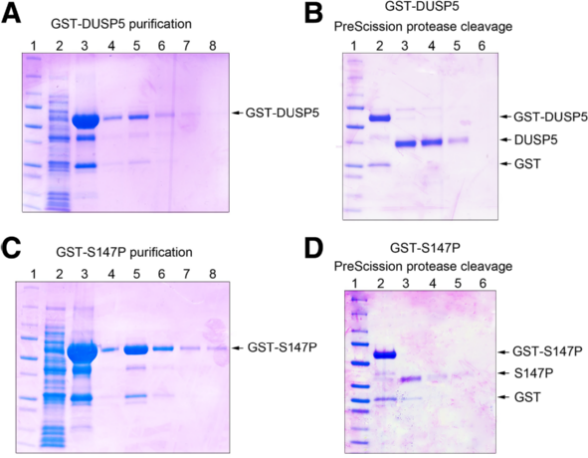
**GST-DUSP5 and GST-S147P Proteins Purification. (A)** GST-DUSP5 protein purification. Lane 1: MW marker, lane 2: cell lysate, lane 3: GST pull down (GST-DUSP5 was adsorbed on glutathione sepharose 4B and loaded on gel), lanes 4–7 purified GST-DUSP5 protein fractions. **(B)** GST-DUSP5 PreScission protease cleavage. Lane 1: MW marker, lane 2: Purified GST-DUSP5, lanes 3–6: GST-DUSP5 protein eluted following PreScission protease digestion. **(C)** GST-S147P protein purification. Lane 1: MW marker, lane 2: cell lysate, lane 3: GST pull down (GST-S147P was adsorbed on glutathione sepharose 4B and loaded on gel), lanes 4–7 purified GST-S147P protein fractions. **(D)** GST-S147P PreScission protease cleavage. Lane 1: MW marker, lane 2: purified GST-S147P, lane 3–6: protein eluted following PreScission protease digestion.

Based on conditions described earlier, we generated 1 L culture of bacteria, and induced it with IPTG accordingly. The *E.coli* cells were harvested, and the bacterial pellet was resuspended in lysis buffer containing 1% Triton X-100. The soluble protein was collected by sonication followed by centrifugation. Purification of cell extract was carried out using glutathione sepharose 4B affinity chromatography. As shown in Figure 1G, lane 4, the majority of the protein bound to the glutathione-sepharose beads. To elute the bound protein, 20 mM reduced glutathione in buffer A50 was used. A single band of 68 kDa was observed in the Coomassie gel (Figure 2A). Free glutathione was removed by repeated steps of concentration of eluate followed by dilution in buffer A50. The final protein yield for 1 L cell culture was 7.8 mg. To remove the GST tag, we performed a PreScission protease cleavage reaction that recognizes the PreScission protease cleavage site embedded between the GST tag and DUSP5. Following the protease cleavage, a single band of 37 kDa was observed in the Coomassie gel (Figure 2B). Using similar methods to wild type DUSP5, we also expressed, induced and purified GST-S147P protein as shown in figures 2C and D. Taken together, we have successfully expressed, purified, and cleaved the GST tag to generate pure untagged DUSP5 and S147P proteins.

### DUSP5 protein characterization

**Figure 3:**
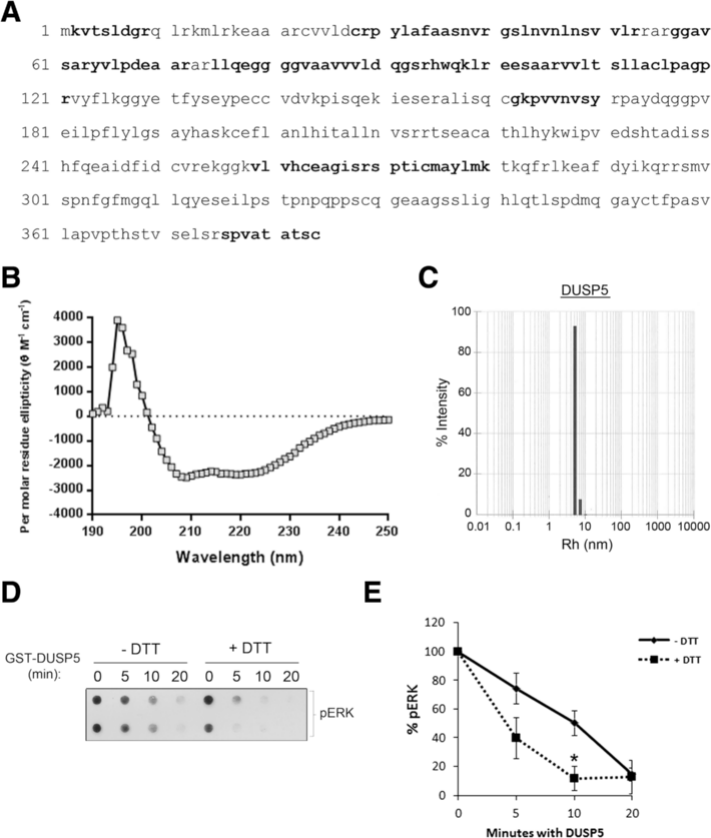
**Protein characterization. (A)** Mass spectrometric analysis was performed by Bioproximity, LLC on coomassie identified bands of the appropriate size separated by SDS-PAGE. Samples were subjected to in-gel trypsin digestion and the peptides recovered for mass spectrometric analysis. **(B)** Circular dichroism was performed to determine folding and secondary structure of DUSP5, which was found to be 43% alpha helical and 20% beta-sheet. **(C)** Dynamic Light Scattering predicts oligomerization of both DUSP5 and S147P. **(D & E)** GST-DUSP5 is active and sensitive to the addition of DTT. Specifically, 1 mM DTT increased the activity of GST-DUSP5 against pERK2 *in vitro*.

To further characterize the proteins, we performed mass spectrometry (LC-MS/MS), dynamic light scattering (DLS), and circular dichroism (CD) analysis. The LC-MS/MS analysis identified peptides corresponding to 35.7% of both DUSP5 and S147P, with 99.7% coverage over the entire protein sequence (Figure 3A and Additional file 3: Figure S1). To test for proper folding of our proteins we utilized CD along with DICHROWEB to estimate secondary structure (Figure 3B and Additional file 4: figure S2). Our CD results indicated that cleaved DUSP5 contained 43% alpha helix and 20% beta sheet. This data tightly correlates with our predicted structure model of DUSP5 (described later) which contains 40.9% alpha helix and 15.6% beta sheet. Additionally, these results correlate to previously reported CD spectra from DUSP6 [23] and similar results were obtained for all DUSP5 constructs. Thus, these results confirm the identity of the purified, cleaved DUSP5 and S147P recombinant proteins and indicate proper folding.

We next performed DLS, which measures fluctuations in scattering intensities emanating from particles that undergo random Brownian motion. Diffusion coefficient and particle size (hydrodynamic radius – Rh) information are determined from analyzing these fluctuations. When DLS was performed on both wild type DUSP5 and S147P proteins in solution, we observed trace amounts of very large molecular weight species (Rh > 30 nm) (Figure 3C and Additional file 5: figure S3). The main protein peak is spread around 5 nm, and the polydispersity varies as well. The estimated Rh range for DUSP-5: 3.6 to 6.0; average (n = 7) Rh = 4.8 nm which corresponds to a MW of 132 kDa. For S147, the estimated Rh ranged from 3.3 to 5.3; average (of seven measurements) Rh = 4.5 nm which corresponds to a MW of 114 kDa. The predicted molecular weight of the protein is 42 kDa, and DUSP5 protein in a dimer configuration would be 84 kDa. A typical Rh value of 4.0 nm would correspond to about 86 kDa. Because the crystal structure of the C-terminal phosphatase domain is reported to form a dimer (PDB code, 2g6z), we investigated whether disulfide formation between cysteine residues in the proteins would contribute to this dimer formation and accordingly activity of these proteins. Addition of DTT showed a clear increase in the phosphatase activity of the WT DUSP5 protein (Figure 3D). The DLS results and the DTT results point to possible association of protomers, likely dimers, being the dominant species in solution for both wild type and S147P proteins, as well as sensitivity to reducing environments. Taking the protein characterization data together, we have successfully generated full-length, soluble, folded versions of both GST-DUSP5 and GST-S147P proteins and their cleaved counterparts from bacteria.

### Activity of GST-tagged and untagged DUSP5 proteins

**Figure 4:**
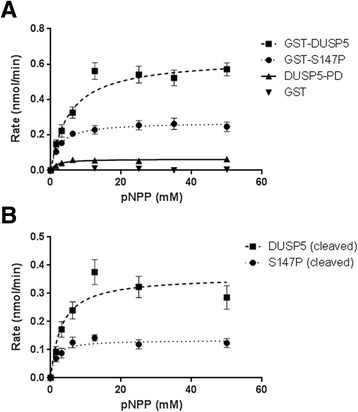
**GST-DUSP5 and GST-S147P proteins are active against pNPP in vitro. Panels (A-B)** are Michaelis-Menten curves derived from pNPP assays performed with recombinant proteins. **(A)** Michaelis-Menten curves with GST, GST-DUSP5 and GST-S147P proteins. **(B)** Michaelis-Menten curves with cleaved versions of S147P and DUSP5 proteins.

To determine the activity of GST-tagged proteins, we performed para-nitrophenol phosphate (pNPP) activity assay (Figure 4). In this assay, 1 μM of recombinant DUSP5 proteins was added to assay buffer, and the substrate, pNPP, was added last to the wells at the indicated concentrations. Absorbance was measured at 405 nm every 30 seconds for 5 min, and the ΔAbs/Δt used to calculate initial velocities, which were then plotted as Michaelis-Menten curves and fitted (nonlinear least squares regression). As expected, the GST protein had no activity in this assay. Interestingly, the mutant exhibited a 2-fold decrease in V_max_ when compared to the wild type DUSP5 protein. The GST-S147P exhibited V_max_ = 0.27 ± 0.01 nmol/min and K_m_ = 2.3 ± 0.56 mM, while GST-DUSP5 exhibited V_max_ = 0.63 ± 0.03 nmol/min and K_m_ = 5.23 ± 1.0 mM (Figure 4A and Table 1). We also obtained a His-tagged DUSP5-PD domain-only protein from our collaborators, which was similar to the one crystallized by Jeong et al. [6], and investigated its activity in the pNPP assay in comparison to the two full-length DUSP5 proteins. Interestingly, the PD alone had lower V_max_ (0.06 ± 0.003 nmol/min) and K_m_ (1.7 ± 0.45 mM) than GST-DUSP5 or GST-S147P full-length proteins, implying that the EBD domain is important for DUSP5’s activity (Table 1). We next investigated the activity of the DUSP5 protein which was cleaved of its GST tag (Figure 4B). The cleaved WT DUSP5 shows lower V_max_ = 0.36 ± 0.03 nmol/min and K_m_ = 3.0 ± 1.0 mM compared to GST-DUSP5, and similarly cleaved S147P shows lower V_max_ = 0.13 ± 0.01 nmol/min and K_m_ = 1.3 ± 0.55 mM compared to GST-S147P (Figure 4B and Table 1). However, the trend of S147P showing decreased activity compared to DUSP5 is consistent for both cleaved and uncleaved proteins. These results collectively suggest that mutant S147P protein is less active than wild type DUSP5 protein and that both EBD and PD domains are necessary for full activity of DUSP5.

**Table 1:** **Michaelis-Menten parameters (Non-linear least squares regression)**

***M-M Kinetics***	**GST-DUSP5**	**GST-S147P**	**GST**	**DUSP5-PD**
**Vmax (nmol/min)**	0.63 ± 0.03	0.27 ± 0.01	NA	0.06 ± 0.01
**Km (mM)**	5.24 ± 1.0	2.33 ± 0.56	NA	1.7 ± 0.45
***M-M Kinetics***	**DUSP5 (cleaved)**	**S147P (cleaved)**		
**Vmax (nmol/min)**	0.36 ± 0.03	0.13 ± 0.01		
**Km (mM)**	3.0 ± 1.0	1.3 ± 0.55		

### Activity of the mutant S147P and WT DUSP5 to pERK

**Figure 5:**
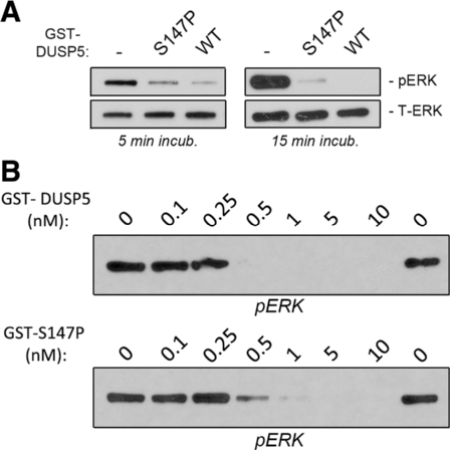
**GST-DUSP5 and GST-S147P proteins are active against pERK in vitro.** Panels **(A-B)** are p-ERK western blots performed with purified WT DUSP5 or S147P proteins. **(A)** Time course experiment with 5 min and 15 min incubation of DUSP5 recombinant proteins with pERK. **(B)** Dose curve of recombinant DUSP5 proteins with a 15 min incubation period with pERK.

We complemented the pNPP assay with a second assay using the natural substrate of DUSP5, pERK2 (Figure 5). In this assay, GST-DUSP5 and GST-S147P (0.5 nM) are incubated in the presence of purified pERK2 (10 nM) for 5 or 15 min, and dephosphorylation determined by western blot. As shown in Figure 5A, incubation with GST-DUSP5 protein decreases pERK levels more than GST-S147P at both time points indicating hypoactivity of S147P to its natural substrate, pERK, and corroborating the hypoactivity of S147P observed in the pNPP activity assay. We also performed a titration curve with different doses (0.1-10 nM) of GST-DUSP5 and GST-S147P proteins in the pERK assay (Figure 5B). At 0.5 nM concentration of GST-DUSP5, the pERK band is completely absent, and the comparable concentration for the same effect for GST-S147P is 5 nM, a ten-fold higher concentration (Figure 5B). Again, these data along with the pNPP assay data suggest that S147P is less active than WT DUSP5 protein.

### Modeling studies

To further explain the mechanism of the hypoactivity of mutant S147P protein, we performed computational modeling studies. The complete model of DUSP5/pERK2 was built using two domains of DUSP5, i.e. ERK-binding domain (EBD) and phosphatase domain (PD), and phosphorylated ERK2. The structure of PD was available from the X-ray crystallography [6], while the structure of EBD was obtained by homology modeling (see Experimental Procedures section). The crystal structure of non-phosphorylated ERK2 [24] was modified by insertion of the phosphate groups in the Thr185 and Tyr187 residues, and it was refined by a long (8 ns) molecular dynamic simulation, during which the phosphorylated threonine (TPO) and tyrosine (PTR) residues adopted conformations similar to those reported for rat pERK2 [1] (Additional file 6: Figure S6).

The initial assembly of PD with pERK2 was guided by the fact that the X-ray structure of PD contained two sulfate ions, which were separated by ~7 Å [6], and therefore it was surmised that the phosphate groups of TPO185 and PTR187 of pERK2 occupy these same sites in the complex of PD with pERK2. Indeed, a juxtaposition of these domains using the YASARA software [13] resulted in a structure, in which the phosphate group of PTR187 penetrated deep inside the binding site of the active center of PD, and stability of the PD/pERK2 complex was confirmed by 8-ns MD simulations. Next, the Z-Dock protein-protein docking server [25] was utilized to juxtapose EBD to pERK2 while only taking into account the proximity of the kinase interaction motif of EBD (^52^LRR^54^) [5] and common docking motif (^318^DPSD^321^) of pERK2 [26]. The resulting structure showed not only the interaction between the requested motifs but also predicted interactions of a hydrophobic groove near the common docking motif in pERK2 [26] with the hydrophobic segment of EBD (^59^AVSA^62^), which is likely to be important for the specificity of DUSP5 towards ERK [26]. Furthermore, our confidence in the model was supported by the fact that the position of EBD in the predicted structure matches a large spot of highly conserved residues on the back face of pERK2 (Additional file 7: Figure S7). The resulting structure of the EBD/pERK2/PD complex was refined by a 500 ps-long MD simulation.

**Figure 6:**
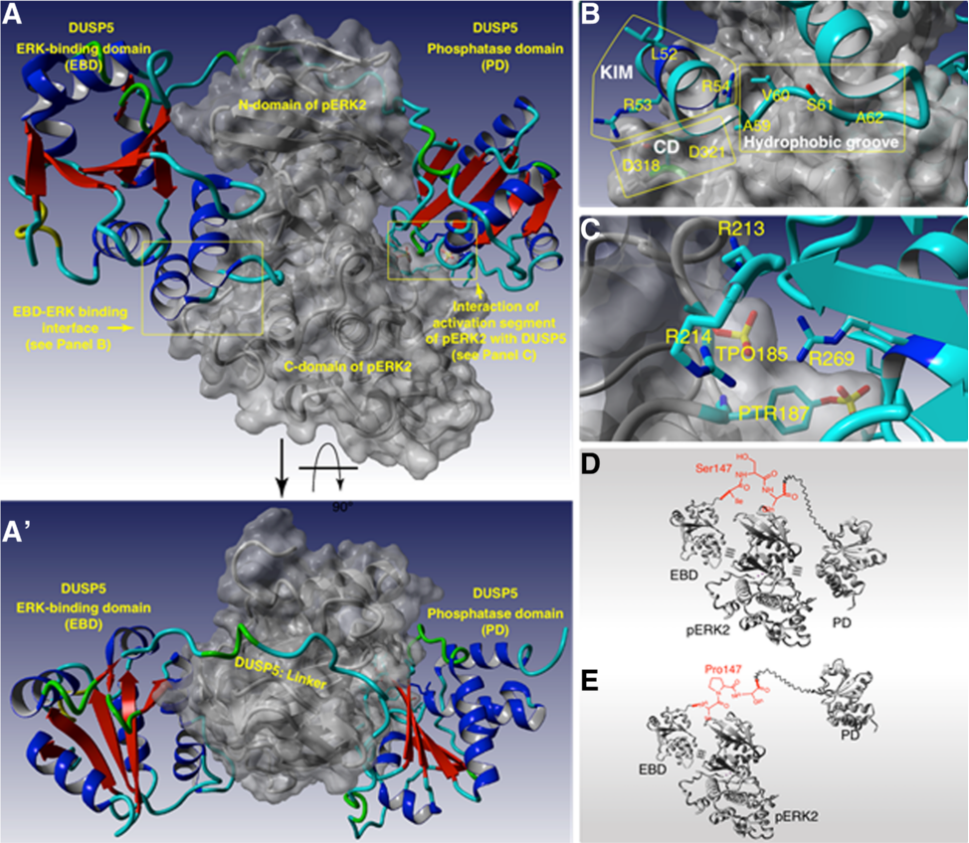
**Computational modeling of human pERK2 and DUSP5 proteins. Panels (A and A’)** are computational model of human pERK2 with DUSP5 protein complex from different points of view (pERK2 is shown in surface representation). **Panel (B)** represents the binding interface between EBD and pERK2, which consists of kinase interaction motif (KIM) of EBD and common docking motif (CD) of pERK2, as well as of hydrophobic 59AVSA62 sequence of EBD and hydrophobic groove of pERK2. **Panel (C)** represents the interaction of activation segment of pERK2 (TPO185-Glu-PTR187) with the active site of DUSP5. **Panels (C and D)** are schematic representation of the effect of serine-to-proline mutation on the DUSP5/pERK2 complex.

Next, we connected the C-terminus of the EBD to the N-terminus of the PD in the EBD/pERK2/PD complex by the linker, which consists of ~40 amino acids (Figure 6A and A’). The position of the N-terminus of the linker corresponds to one end of a deep and long groove on top of the N-domain of pERK2, which suggests a possible pathway around pERK2. Indeed, after arranging the linker in the groove on the surface of N-domain of pERK2 and refining the structure by an 8-ns MD simulation (Figure 6A and A’), we found that the linker not only spans the needed length but is also involved in a number of favorable interactions with pERK2 (e.g. the interacting pairs of residues of DUSP5 and pERK2: Ile146–Val46, Glu154–Gln97, Arg155–Asp20, Ser159–Asp100), which facilitate effective binding.

Based on this model, we propose the following scenario of DUSP5 binding to pERK2. First, EBD binds to the C-domain of pERK2, which brings the N-terminal side of the DUSP5 linker to the beginning of the groove on top of the N-domain of pERK2 (Figure 6B). Next, the linker arranges itself in this groove in a zipper-like action, thereby bringing PD to the phosphorylated residues of pERK2. Finally, the two anion-binding sites of PD recognize the phosphorylated threonine and tyrosine residues of pERK2 that are present in the pThr-Glu-pTyr region of its activation loop, directing insertion of PTR187 into the active site of DUSP5 (Figure 6C), where the trans-phosphorylation or dephosphorylation reaction occurs.

To analyze the effect of the S147P mutation, we swapped Ser147 to proline in the model of the DUSP5/pERK2 complex, and performed an 8-ns molecular dynamic simulation. Analysis of the MD trajectories showed that the mutation leads to structural rearrangement in DUSP5, which involves displacement of the EBD and N-terminal side of the linker away from the N-domain of pERK2 (Additional file 8: Figure S8). These distortions indicate that mutation of Ser147 to proline in the DUSP5 leads to an altered arrangement of the linker in the groove of pERK2. Thus, we suggest that the observed reduction in the catalytic activity of the mutant S147P originates because of deviation from the optimal binding of the EBD-PD linker to pERK2, thereby resulting in a slower formation of pre-reaction complex and slower rate. Indeed, based on the cursory examination it is expected that the conformational changes in the linker arising from serine-to-proline mutation should alter the optimal arrangement of the linker in the binding groove of pERK2 (Figure 6D and E). Collectively, the molecular simulation studies provide a molecular explanation for the decreased activity of S147P towards pERK when compared to wild type DUSP5 protein.

## Discussion

This study defines and describes the generation of an active protein of clinical relevance, namely DUSP5, in milligram amounts that allows for future structure-function based studies. Importantly, we describe the soluble production of full length GST-tagged DUSP5 and mutant S147P proteins that are active. We also demonstrate that the S147P protein is less active than wild type DUSP5 in its activity towards two distinct substrates and that the two domains of DUSP5 are critical for its full functional activity. Lastly, we provide for the first time a computational structure analysis of the ERK-DUSP5 interaction, from which we identify a potential active role of the DUSP5 linker region in ERK binding. Our structural analysis also reveals a potential mechanism to the reduced activity of the S147P protein.

Previously, DUSP5 protein has been crystallized, and the source of this protein was from *E.coli* [6]. However, this crystal structure was determined from the PD alone, and the authors also included a mutation Cys263Ser, which was likely necessary to obtain well-diffracting crystals. We rationalized that in order to study the complete structural conformation of DUSP5 and mutant DUSP5, milligram amounts of the proteins was needed in a soluble form. We utilized GST to tag DUSP5, since GST is known to facilitate the folding and solubility of proteins [27]. GST-DUSP5 protein clearly was soluble, and our yields routinely ranged in the 5–10 mgs per liter concentration of bacterial culture thereby suggesting that future structure studies are feasible with scale-up. Due to the bulky nature of GST (26 kDa) in relation to the DUSP5 protein size (42 kDa), we cleaved the GST off the DUSP5 using PreScission protease. This introduced two issues: (a) multiple bands were observed on PreScission cleavage, and (b) the yield dropped significantly. We sequenced the bands resulting from the PreScission cleavage reactions, and confirmed that one of the bands was indeed cleaved DUSP5 protein. Although yields are low, we can scale-up the cultures to overcome this limitation of the cleaved protein. Because the crystal structure of the C-terminal PD is a dimer (PDB code, 2g6z) [6], it is plausible that the C-terminal PD domain of the full length protein do interact to form a dimer in solution. Based on the DLS data, if there is any interaction between the N-terminal domains, the hydrodynamic radius (Rh) could be higher than expected for a “strict” dimer. Rh would be less if both N- and C- domains of two protomers interact with the respective partners. Conversely, Rh would be high if only the C-terminal domains form dimeric interaction while the N-terminal domains are only coming along because they are attached. In either scenario, the propensity of DUSP5 to form oligomers is a property that appears to be functionally relevant since breaking disulfide bonds with DTT, and thus presumably creating single, monomeric species, altered enzymatic activity of the protein. Whether the mutation in DUSP5 has a propensity to oligomerize is a question that needs further investigation.

pERK2, the natural substrate for DUSP5, is well established in its role in endothelial cell proliferation [28]. The present model for binding [29] has the two domains (EBD & PD) of DUSP5 enveloping pERK2 on opposite faces as shown in Figure 6A/A’ in a tweezer-like action. Based on analogy to other MAP kinase phosphatases [29], we expect that the EBD and PD domains of DUSP5 bind ERK cooperatively, such that the previously reported ERK binding motif in the EBD [29] (^52^LRR^54^, Figure 6B) interact with the common docking motif (^318^DPSD^321^) of pERK2 (Figure 6B), and the hydrophobic segment in EBD (^59^AVSA^62^) interacts with the hydrophobic groove of pERK2, as shown on Figure 6B. Previously, Camps et al. [30] demonstrated that ERK binding to the non-catalytic amino-terminal domain of MKP3 dramatically activates the phosphatase catalytic domain. Structural and physical evidence suggests that ERK activates MKP3 through the stabilization of the active phosphatase conformation, inducing closure of the catalytic “general acid” loop [31]. In this closed conformation, the loop structure can participate efficiently in general acid/base catalysis, substrate binding, and transition-state stabilization. However, catalytic activity of DUSP5 PD does not seem to require activation through binding of its substrate, pERK [5,6], and therefore the inhibition observed due to S147P mutation must arise from the structural rearrangement of the EBD–PD linker in the DUSP5/pERK2 complex. Based on our molecular modeling studies, the linker in the WT DUSP5/pERK2 complex is aligned in the groove on top of the N-terminal domain of pERK2 (Figure 6A/A’). The S147P mutation disrupts the optimal alignment of the linker in the pERK2 groove due to the dramatic conformational change (Figure 6D/E) and thus results in a slower formation of the *pre*-reaction complex and reduced catalytic activity of S147P mutant. Deviation from the optimal binding of DUSP5 to pERK could result into following consequences. First, the S147P mutation can destabilize a *pre*-reactive complex to facilitate its dissociation to the non-interacting DUSP5 and pERK, which would lead to a further reduction in the overall reaction rate. Second, the S147P mutation can as well facilitate decomposition of the *post*-reaction complex. We believe that the latter process is not a rate-limiting reaction step either in WT or mutant DUSP5 complex, which is in agreement with the recent theoretical study of trans-phosphorylation in Cdc25 [32], and therefore it should not affect the overall reaction rate. However, we plan to address this question in greater detail in our future planned theoretical study.

We recreated the previously identified S147P mutation in patients with vascular anomalies [3] in the GST-DUSP5 template, and successfully generated milligram amounts of soluble S147P protein. Until now, it was not known whether this mutation was a gain- or loss-of-function. Our assays here imply that S147P is a loss-of-function mutation in that it dephosphorylates residues in pNPP or pERK at a slower rate than wild type DUSP5 protein; however, the physiological relevance of this drop in activity *in* vitro remains to be determined. If this mutation is partially responsible for the natural history of vascular anomalies, it implies that the loss-of-function mutation is likely to slow the removal of phosphates from active pERK. Since ERK activity is often correlated with the cells ability to proliferate, such as the case in cancer [33,34], the endothelial or vascular progenitor cell that contains the causative DUSP5 somatic mutation is likely to lose the ability to negatively regulate pERK, thus causing endothelial cell to hyperproliferate, and display the vascular anomaly phenotype. Besides vascular anomalies, DUSP5 has also been implicated as a target for cardiac hypertrophy and immune conditions. Recent data suggests that small molecule histone deacetylase (HDAC) inhibitors suppress cardiac hypertrophy via inhibition of DUSP5 [35]. Cardiomyocytes, the primary cell type involved in cardiac hypertrophy, show differential effects on ERK signaling in the nucleus and cytoplasm; in that nuclear ERK1/2 activation is suppressed in a DUSP5-dependent manner, while cytosolic ERK1/2 activation is maintained under these same conditions [35]. In regards to the role of DUSP5 in immunology, DUSP5 expression is induced by IL-2 stimulation and regulates B-cell differentiation [36]. Additionally, overexpression of human DUSP5 in mice results in an autoimmune phenotype, as well as increased proliferation of activated memory T-cells [37]. Therefore, finding small molecule that selectively activate or inhibit DUSP5 in a context-dependent manner is important, and is the focus of studies in our laboratories.

## Conclusions

DUSP5 has been shown to be a critical protein involved in several disease phenotypes. Therefore, to better understand the structure-function relationship of DUSP5, we generated and characterized both wild-type and mutant DUSP5 while providing a comprehensive molecular model of the DUSP5/ERK interaction. In addition to showing decreased activity of the mutant DUSP5, our molecular model revealed a novel role for the DUSP5 linker region in DUSP5/ERK interactions. Taken together, our studies will allow for the identification of activators or inhibitors of DUSP5; as well as the selective modulation of S147P’s activity rather than wild type DUSP5 activity – a concept that is the hallmark of targeted drug discovery approaches.

## Availability of supporting data

The data sets supporting the results of this article are included within the article and its additional files.

## Additional files

Additional file 1: Figure S4. Molecular modeling of DUSP5 EBD. The molecular surface (left side) and cartoon & sticks representation (right side) of the hybrid model of DUSP5 EBD colored by the residue conservation scores obtained from the ConSurf Server [38] (web address: consurf.tau.ac.il). Cyan color corresponds to most variable residues, violet color—to the most conserved ones. Additional file 2: Figure S5 Homology model validation. Validation of the homology model based on the 1HZM template (top) and the hybrid model (bottom) by the SAVES server (left side) and PROSESS server (right side). Please visit http://services.mbi.ucla.edu/SAVES/T/?job=16119 and http://services.mbi.ucla.edu/SAVES/T/?job=16120 for the full HTML reports from the SAVES server. Additional file 3: Figure S1. Mass spectrometry of mutant DUSP5. The cleaved, purified mutant DUSP5 (S147P) was sent to Bioproximity for mass spectrometric protein identification. Mass spectrometric analysis was performed on Coomassie identified bands of the appropriate size separated by SDS-PAGE. Samples were subjected to in-gel trypsin digestion and the peptides recovered for mass spectrometric analysis, LC-MS/MS. Mass spectrometric analysis performed on the peptide fragments identified 35.7% of the DUSP5 sequence (bold), with 99.7% coverage over the entire protein sequence for both WT DUSP5 and S147P. Additional file 4: Figure S2. Circular dichroism data with WT DUSP5 and mutant constructs. Folding and secondary structure composition of the recombinant DUSP5 constructs was assessed by circular dichroism (CD). The spectra were deconvoluted using DICHROWEB. Secondary structure determinations were as follows: GST-DUSP5 34% α-helix and 19% β-sheet; GST-S147P 31% α-helix and 11% β-sheet; DUSP5 43% α-helix and 20% β-sheet; S147P 58% α-helix and 8% β-sheet. The normalized root-mean-square deviations (NRMSD) were less than 0.1 for all constructs. Additional file 5 Figure S3. Dynamic Light Scattering of S147P. When DLS was performed on both native DUSP-5 and S147P proteins in solution, we observed trace amounts of very large molecular weight species (Rh > 30 nm). For S147, the estimated Rh ranged from 3.3 to 5.3; average (of seven measurements) Rh = 4.5 nm which corresponds to a MW of 114 kDa. This data indicates that S147P, normally 42 kDa, may dimerize in solution. Additional file 6: Figure S6. Phosphorylated ERK2 modeling. Top: structure of human pERK2 after manual addition of phosphate groups and 500-ps molecular dynamic refinement. Bottom: crystal structure of rat pERK2 (PDB: 2ERK [1]). Additional file 7: Figure S7. ERK2 molecular surface. The molecular surface of ERK2 (PDB code: 2OJG [24]) colored by the residue conservation scores obtained from the ConSurf Server [38] (web address: consurf.tau.ac.il). Cyan color corresponds to most variable residues, violet color—to the most conserved ones. Encircled is the sulfate anion found in the X-ray structure of dephosphorylated ERK. The conserved residues on the left panel are responsible for the kinase activity of pERK2 as well as interactions with phosphatase domains of DUSPs (N-domain of pERK2 is on the upper side of the figure, while N-domain – on the lower side). Compact localization of conserved residues on the rear side of pERK2 (left panel) presumably denotes interface for binding with EBDs. Additional file 8: Figure S8. Juxtaposed model structures of WT and mutant DUSP5. The molecular representation of the juxtaposed model structures of WT (blue color) and S147P mutant (green color) DUSP5. Blue/green ellipses denote orientation of individual domains. Yellow rectangle highlights structural displacement near the N-side of the EBD-PD linker; curved yellow arrow denotes arrangement of the resting part of the linker.

## Abbreviations

CD: Circular dichroism
DLS: Dynamic light scattering
DTT: Dithiothreitol
DUSP: Dual specificity phosphatase
EBD: Erk binding domain
ERK: Extracellular-regulated kinase
GST: Glutathione-S-transferase
HDAC: Histone deacetylase
IPTG: Isopropyl β-D-1-thiogalactopyranoside
JNK: c-Jun N-terminal kinases
LB: Lysogeny broth
MAPK: Mitogen-activated protein kinase
MD: Molecular dynamic simulation
M-M: Michaelis-Menten
MW: Molecular Weight
PD: Phosphatase domain
pERK: phosphorylated extracellular-regulated kinase
pNPP: para-Nitrophenylphosphate
PTR: Phosphorylated tyrosine
S147P: Serine 147 to proline mutant
TB: Terrific broth
TPO: Phosphorylated threonine
WT: Wild-type
