# Supplementary figures and images for: Protein expression, characterization and activity comparisons of wild type and mutant DUSP5 proteins
Source: BMC Biochem. 2014 Dec 18;15:27. doi: 10.1186/s12858-014-0027-0 (PMC4299175; doi:10.1186/s12858-014-0027-0)

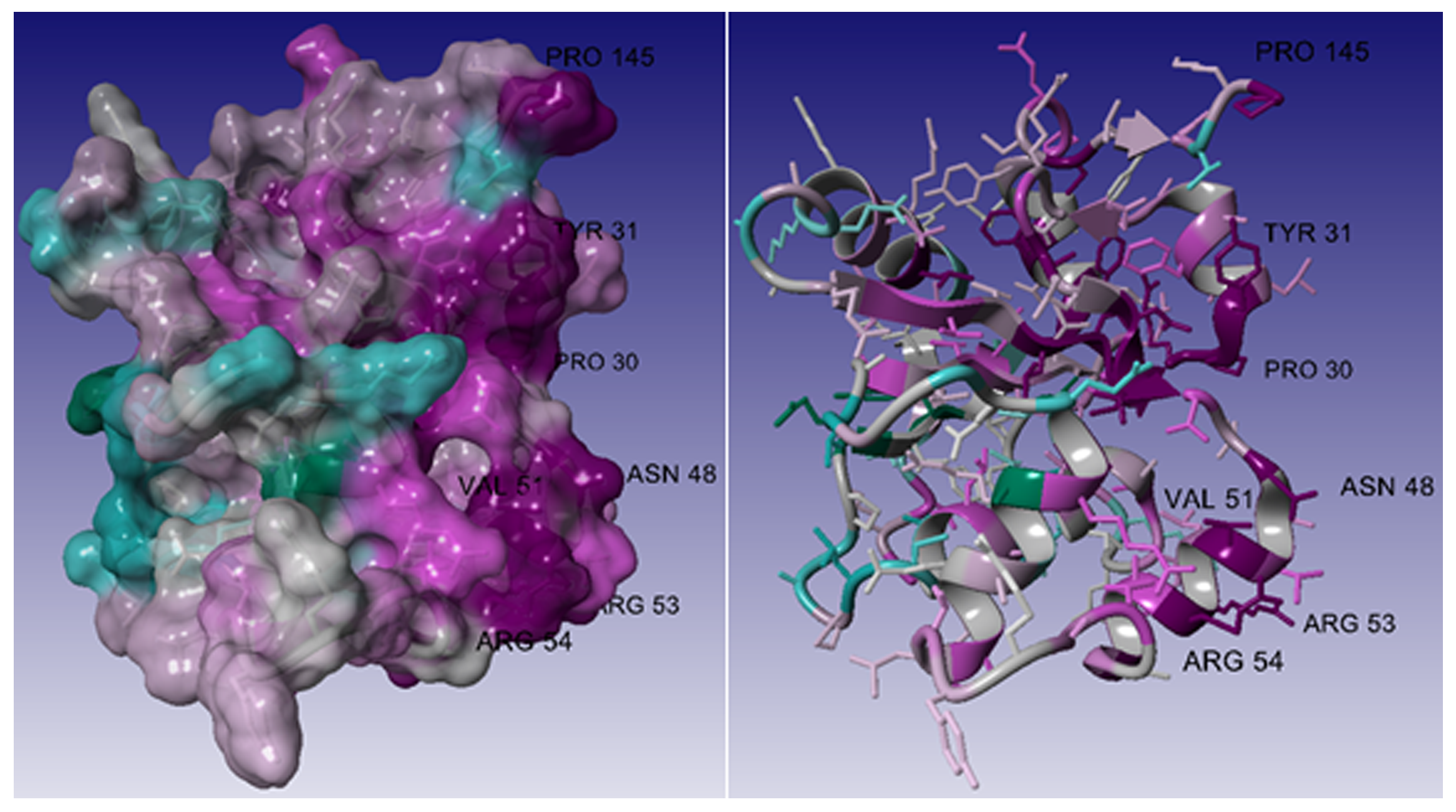

Supplement: Additional file 1: Figure S4. — Molecular modeling of DUSP5 EBD. The molecular surface (left side) and cartoon & sticks representation (right side) of the hybrid model of DUSP5 EBD colored by the residue conservation scores obtained from the ConSurf Server [38] (web address: consurf.tau.ac.il). Cyan color corresponds to most variable residues, violet color—to the most conserved ones. [file 12858_2014_27_MOESM1_ESM.tiff]

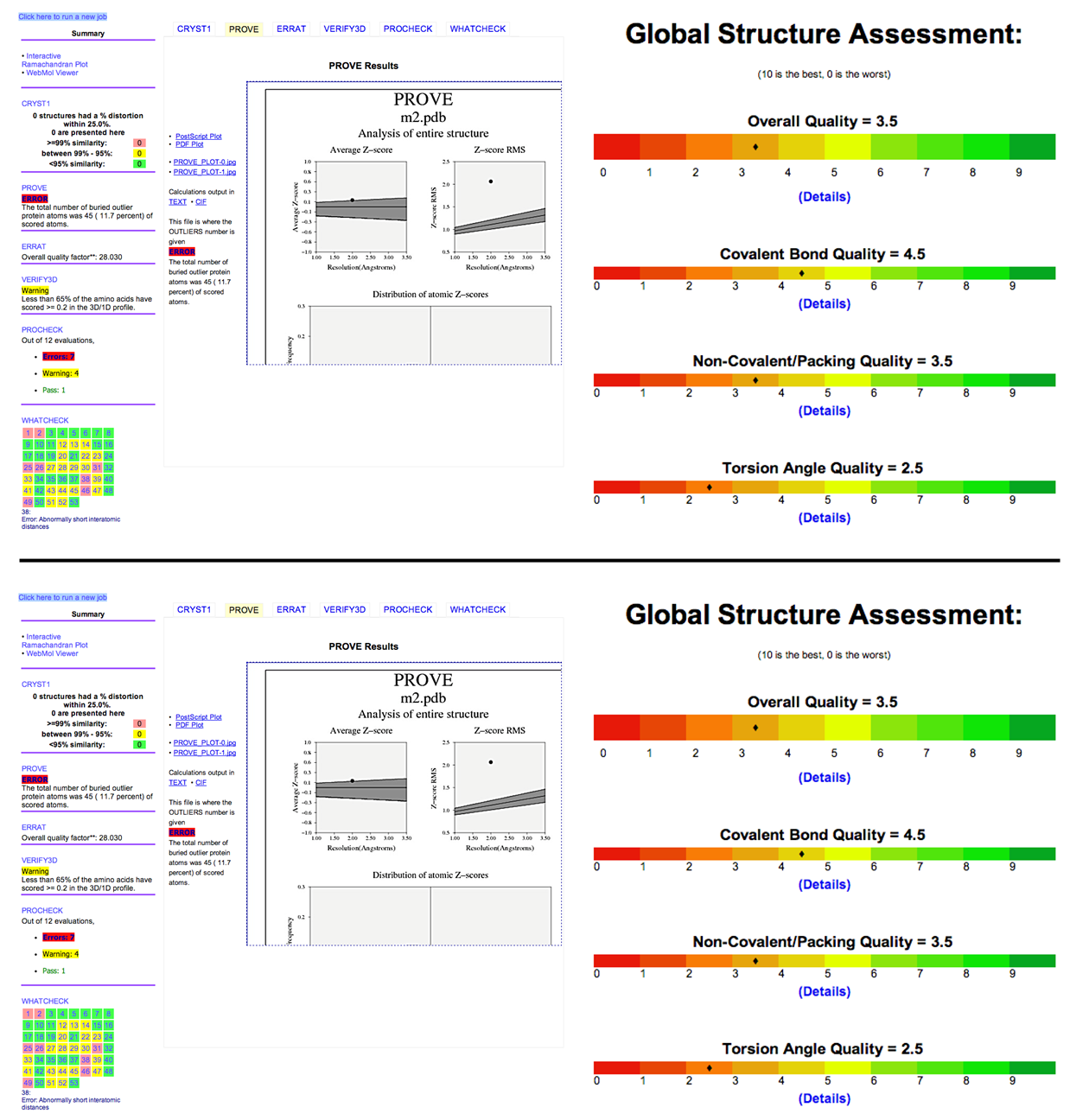

Supplement: Additional file 2: Figure S5 — Homology model validation. Validation of the homology model based on the 1HZM template (top) and the hybrid model (bottom) by the SAVES server (left side) and PROSESS server (right side). Please visit http://services.mbi.ucla.edu/SAVES/T/?job=16119 and http://services.mbi.ucla.edu/SAVES/T/?job=16120 for the full HTML reports from the SAVES server. [file 12858_2014_27_MOESM2_ESM.tiff]

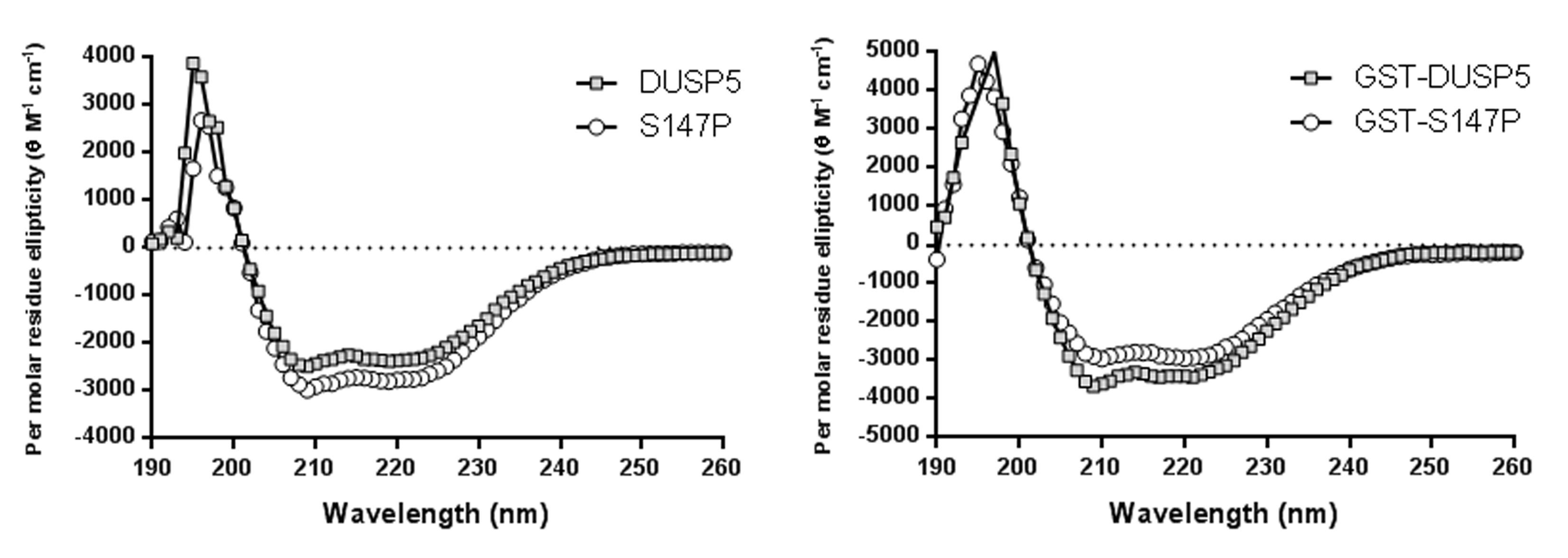

Supplement: Additional file 3: Figure S1. — Mass spectrometry of mutant DUSP5. The cleaved, purified mutant DUSP5 (S147P) was sent to Bioproximity for mass spectrometric protein identification. Mass spectrometric analysis was performed on Coomassie identified bands of the appropriate size separated by SDS-PAGE. Samples were subjected to in-gel trypsin digestion and the peptides recovered for mass spectrometric analysis, LC-MS/MS. Mass spectrometric analysis performed on the peptide fragments identified 35.7% of the DUSP5 sequence (bold), with 99.7% coverage over the entire protein sequence for both WT DUSP5 and S147P. [file 12858_2014_27_MOESM3_ESM.tiff]

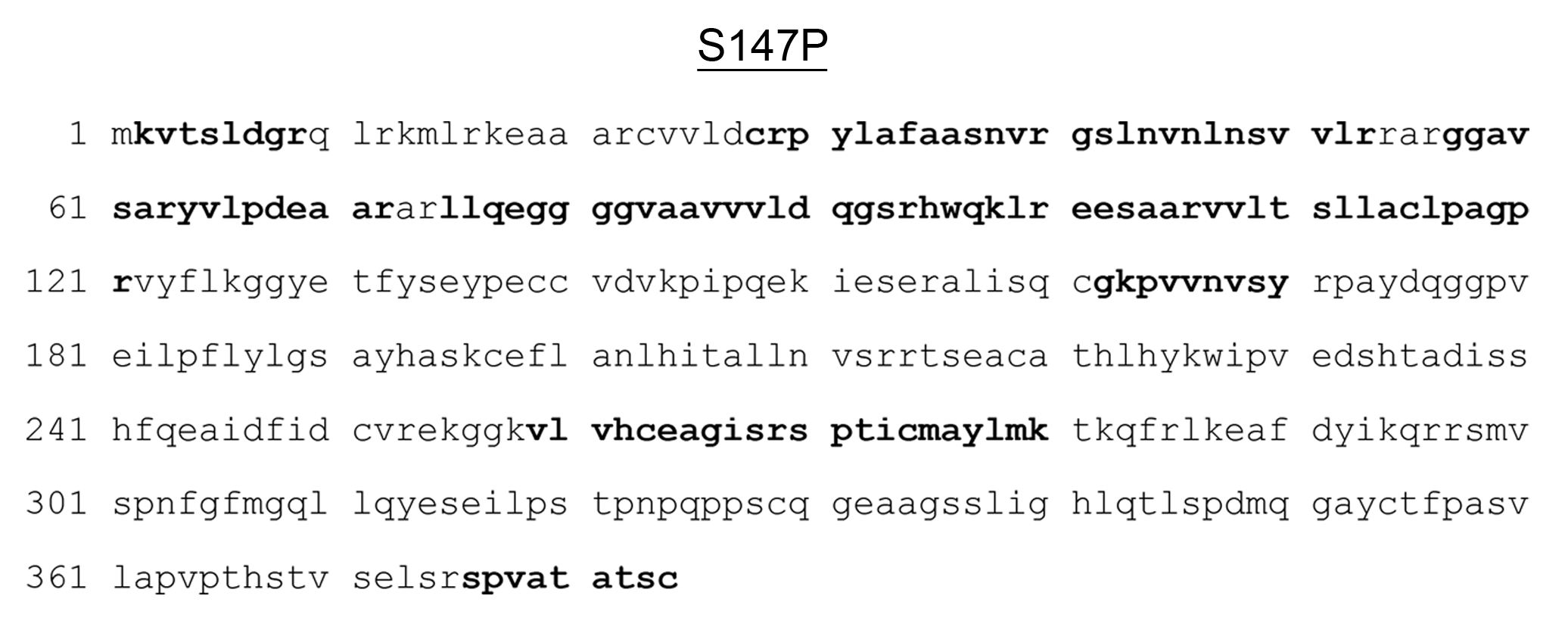

Supplement: Additional file 4: Figure S2. — Circular dichroism data with WT DUSP5 and mutant constructs. Folding and secondary structure composition of the recombinant DUSP5 constructs was assessed by circular dichroism (CD). The spectra were deconvoluted using DICHROWEB. Secondary structure determinations were as follows: GST-DUSP5 34% α-helix and 19% β-sheet; GST-S147P 31% α-helix and 11% β-sheet; DUSP5 43% α-helix and 20% β-sheet; S147P 58% α-helix and 8% β-sheet. The normalized root-mean-square deviations (NRMSD) were less than 0.1 for all constructs. [file 12858_2014_27_MOESM4_ESM.tiff]

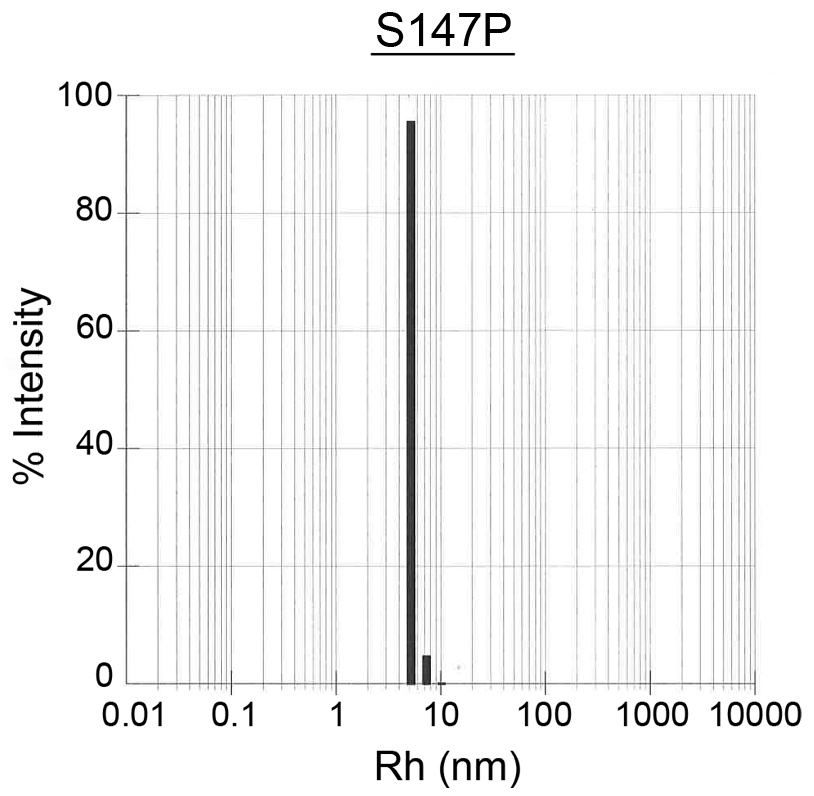

Supplement: Additional file 5 Figure S3. — Dynamic Light Scattering of S147P. When DLS was performed on both native DUSP-5 and S147P proteins in solution, we observed trace amounts of very large molecular weight species (Rh > 30 nm). For S147, the estimated Rh ranged from 3.3 to 5.3; average (of seven measurements) Rh = 4.5 nm which corresponds to a MW of 114 kDa. This data indicates that S147P, normally 42 kDa, may dimerize in solution. [file 12858_2014_27_MOESM5_ESM.tiff]

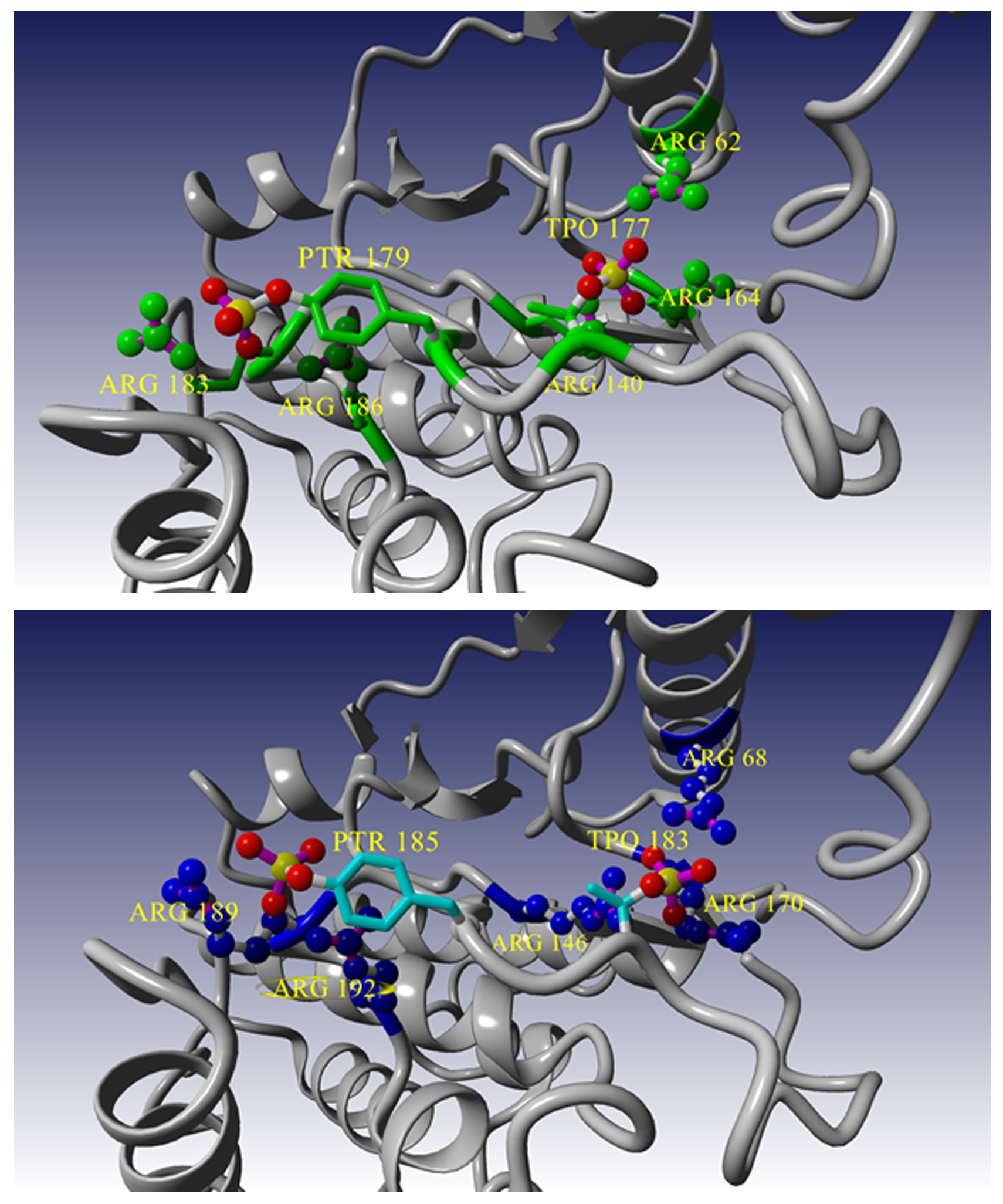

Supplement: Additional file 6: Figure S6. — Phosphorylated ERK2 modeling. Top: structure of human pERK2 after manual addition of phosphate groups and 500-ps molecular dynamic refinement. Bottom: crystal structure of rat pERK2 (PDB: 2ERK [1]). [file 12858_2014_27_MOESM6_ESM.tiff]

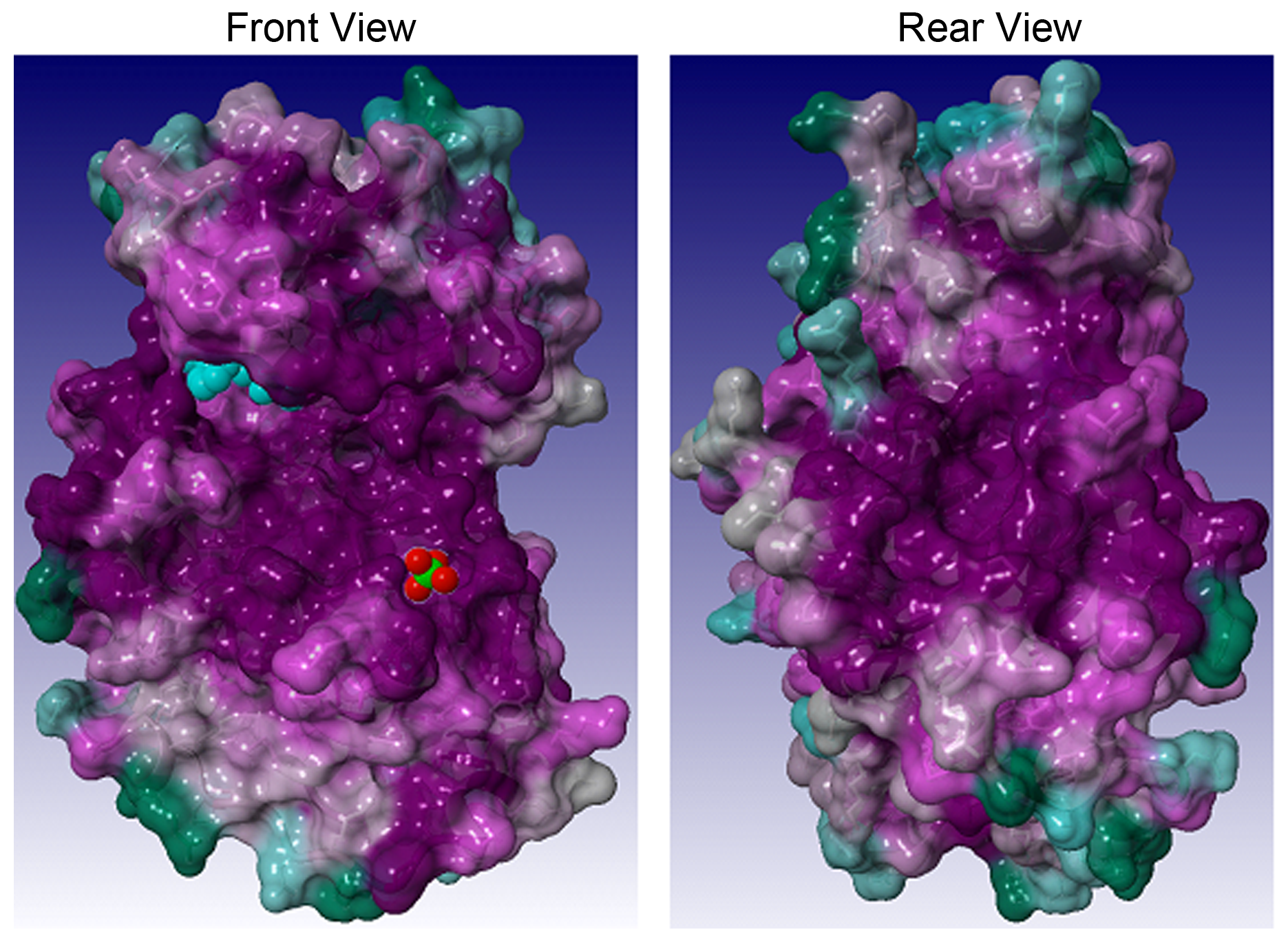

Supplement: Additional file 7: Figure S7. — ERK2 molecular surface. The molecular surface of ERK2 (PDB code: 2OJG [24]) colored by the residue conservation scores obtained from the ConSurf Server [38] (web address: consurf.tau.ac.il). Cyan color corresponds to most variable residues, violet color—to the most conserved ones. Encircled is the sulfate anion found in the X-ray structure of dephosphorylated ERK. The conserved residues on the left panel are responsible for the kinase activity of pERK2 as well as interactions with phosphatase domains of DUSPs (N-domain of pERK2 is on the upper side of the figure, while N-domain – on the lower side). Compact localization of conserved residues on the rear side of pERK2 (left panel) presumably denotes interface for binding with EBDs. [file 12858_2014_27_MOESM7_ESM.tiff]

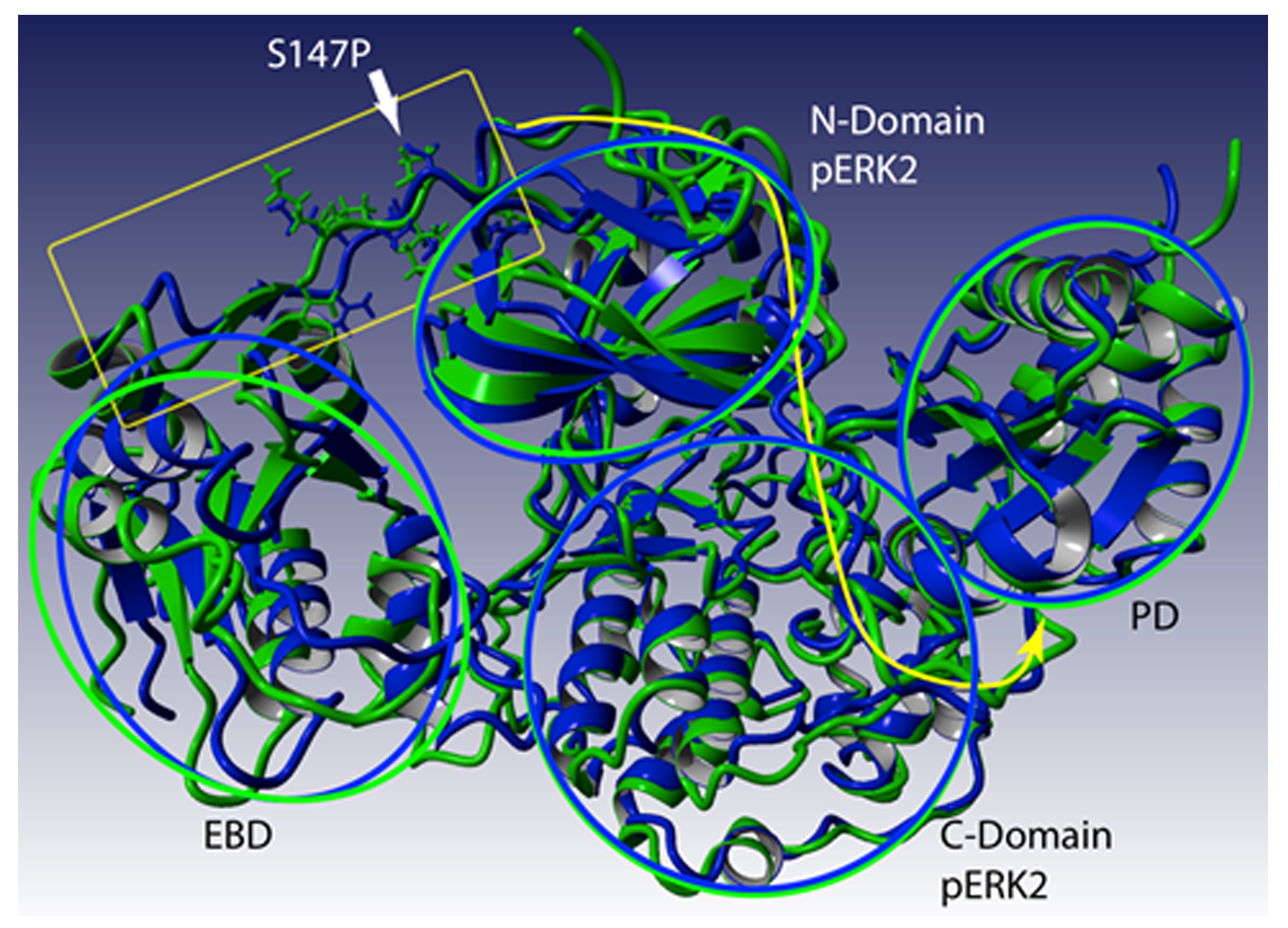

Supplement: Additional file 8: Figure S8. — Juxtaposed model structures of WT and mutant DUSP5. The molecular representation of the juxtaposed model structures of WT (blue color) and S147P mutant (green color) DUSP5. Blue/green ellipses denote orientation of individual domains. Yellow rectangle highlights structural displacement near the N-side of the EBD-PD linker; curved yellow arrow denotes arrangement of the resting part of the linker. [file 12858_2014_27_MOESM8_ESM.tiff]
